# Psilocybin restrains activity-based anorexia in female rats by enhancing cognitive flexibility: contributions from 5-HT1A and 5-HT2A receptor mechanisms

**DOI:** 10.1038/s41380-024-02575-9

**Published:** 2024-04-27

**Authors:** K. Conn, L. K. Milton, K. Huang, H. Munguba, J. Ruuska, M. B. Lemus, E. Greaves, J. Homman-Ludiye, B. J. Oldfield, C. J. Foldi

**Affiliations:** 1 Monash University, Department of Physiology, 26 Innovation Walk, Clayton, VIC 3800 Australia; 2 Monash Biomedicine Discovery Institute, 23 Innovation Walk, Clayton, VIC 3800 Australia; 3 Department of Biochemistry, Weill Cornell Medicine, New York, NY 10065 USA; 4 University of Helsinki, Yliopistonkatu 4, 00100 Helsinki, Finland; 5 Monash Micro Imaging, Monash University, 15 Innovation Walk, Clayton, VIC 3800 Australia

**Keywords:** Neuroscience, Psychology

## Abstract

Psilocybin has shown promise for alleviating symptoms of depression and is currently in clinical trials for the treatment of anorexia nervosa (AN), a condition that is characterised by persistent cognitive inflexibility. Considering that enhanced cognitive flexibility after psilocybin treatment is reported to occur in individuals with depression, it is plausible that psilocybin could improve symptoms of AN by breaking down cognitive inflexibility. A mechanistic understanding of the actions of psilocybin is required to tailor the clinical application of psilocybin to individuals most likely to respond with positive outcomes. This can only be achieved using incisive neurobiological approaches in animal models. Here, we use the activity-based anorexia (ABA) rat model and comprehensively assess aspects of reinforcement learning to show that psilocybin (post-acutely) improves body weight maintenance in female rats and facilitates cognitive flexibility, specifically via improved adaptation to the initial reversal of reward contingencies. Further, we reveal the involvement of signalling through the serotonin (5-HT) 1 A and 5-HT2A receptor subtypes in specific aspects of learning, demonstrating that 5-HT1A antagonism negates the cognitive enhancing effects of psilocybin. Moreover, we show that psilocybin elicits a transient increase and decrease in cortical transcription of these receptors (*Htr2a* and *Htr1a*, respectively), and a further reduction in the abundance of *Htr2a* transcripts in rats exposed to the ABA model. Together, these findings support the hypothesis that psilocybin could ameliorate cognitive inflexibility in the context of AN and highlight a need to better understand the therapeutic mechanisms independent of 5-HT2A receptor binding.

## Introduction

Anorexia nervosa (AN) is characterised by pathological weight loss driven by restrictive feeding and excessive exercise behaviours, has the highest mortality rate of any psychiatric disorder [1], and is the leading cause of death in females aged 15–24 [2]. Life time prevalence rates of AN are estimated at up to 4% in females and 0.5% in males [3] and while selective serotonin reuptake inhibitors (SSRIs) are the leading pharmacological treatment, they do not improve clinical symptoms in underweight individuals with AN [4].Cognitive inflexibility may be a trait marker of vulnerability to AN, considering that dysfunction arises before the onset of symptoms [5] and persists after weight recovery [6]. Impairments in cognitive flexibility have been consistently seen in AN patients [7–10], and are associated with low quality of life [11], making this symptom a primary target for therapeutic intervention. Cognitive flexibility is a fundamental element of executive functioning that allows for behavioural adaptation to a variable environment, and as a consequence, is associated with favourable outcomes throughout the lifespan [12]. This capability is compromised across a range of neuropsychiatric disorders in addition to AN that include but are not limited to; depression and anxiety disorders, substance-use disorders and obsessive-compulsive disorder [13]. In each of these conditions, psilocybin-assisted therapy [14–17] has either been shown to elicit positive outcomes or is being currently trialled.

Converging evidence from clinical trials and preclinical studies indicates that psilocybin is an effective treatment for symptoms of several psychiatric disorders [18] and may circumvent issues with medication compliance because long-term improvements (at least for depression) have been demonstrated after a single dose [14]. However, there is little evidence to date that disentangles its pharmacological efficacy from the clinically-guided psychological intervention that accompanies psilocybin exposure in these trials [19]. Moreover, while the pharmacological actions of psilocybin are now better understood [20, 21], how these actions translate to therapeutic outcomes remains unclear. Based on the proposal that the therapeutic effects of psilocybin relate to the promotion of flexible thinking and relaxation of maladaptive, rigidly held beliefs [14], and the evidence that psilocybin elicits long lasting effects on cognitive and neural flexibility [22], it seems likely that at least some aspects of therapeutic efficacy may be driven by enhanced cognitive flexibility. However, the neurobiological mechanisms through which psilocybin acts to improve cognitive flexibility are unknown, and there are multiple components of learning and cognition that could contribute to enhanced flexible thinking and behaviour after psilocybin treatment that have not been systematically addressed.

There is evidence implicating serotonin (5-HT) dysfunction in AN, with positron emission tomography (PET) imaging studies revealing decreased binding to the 5-HT2A receptor (5-HT2AR) subtype [23] and increased binding to the 5-HT1A receptor (5-HT1AR) subtype [24] in the frontal cortex of patients. Psilocybin is an agonist for both receptor subtypes [25], raising the intriguing possibility that psilocybin could rescue or reverse cognitive inflexibility by re-establishing the balance of 5-HT signalling in those with AN. Whether or not psilocybin has therapeutic effects in individuals with AN will be revealed by ongoing clinical trials (e.g., NCT04052568, NCT04661514, NCT05481736, NCT04505189). However, these trials are not capable of testing the mechanisms through which psilocybin acts to elicit improvements in symptoms; moreover, they have been criticised in recent years for methodological constraints including their inability to blind participants to treatment conditions, which can bias outcomes in line with expectancy effects [26, 27].

Preclinical studies in animal models are critical for advancing the understanding of the behavioural and pharmacological mechanisms underlying the therapeutic effects of psilocybin [28], with evidence converging on increased neuroplasticity as a key driver of beneficial outcomes [29, 30]. Unfortunately, efforts in this space have focused largely on traditional assays of depression-related behaviour in rodents [31], with variable findings of either improvements [32] or no effects [33], dependent on the assay or animal model used [34]. Given the growing appreciation in behavioural neuroscience that these types of behavioural tests (i.e. the forced swim test) do not reliably translate to human depressive syndromes [35] and that reinforcement learning tasks offer key advantages including more relevant clinical links and repeatability [36], these early approaches clearly need to be redressed. Other key methodological details in prior studies need to be considered, particularly the role of multiple dosing (cross-over) designs, antagonising 5-HT2AR with ketanserin (a compound with many known non-serotonergic binding sites [37]), and the measurable motoric side effects of acute psilocybin administration [38].

The investigation of neurochemical or neural circuit substrates of these effects centre around the actions of psilocybin on the serotonin-2 (5-HT2) receptor subtypes [32, 39–43] but the evidence for the role of 5-HT2AR in rodent cognitive flexibility is conflicting, where acute activation either impairs [44] or has no effect on performance [45]. Less attention has been paid to the possibility that actions at other 5-HTRs might mediate cognitive effects of psilocybin, despite 5-HT2A *independent* effects seen for alleviation of depression-like behaviour [32], dendritic spine formation [46], and neuronal synchronicity [47]. It is likely that specific aspects of psilocybin-induced cognitive flexibility involve other 5-HT receptors [48, 49] and their integration with other neuromodulatory systems, most notably dopamine [50–52]. The challenge in identifying the neuronal substrates for improved flexibility after psilocybin is heightened when attempting to understand whether there may be disorder-specific effects in individuals with AN [53, 54], who present with disturbed 5-HT function that remains inadequately understood.

In the present study, our objective was to comprehensively investigate how psilocybin, in a 5-HT receptor-dependent manner, may alter some of the core components that underlie cognitive flexibility, such as incentive motivation and task engagement [55], response inhibition [55], and reward efficacy [56]. All animals received psilocybin only once, with or without prior administration of selective 5-HT1A and 5-HT2A receptor antagonists, and learning outcomes were assessed post-acutely. In addition, we used the most well-established rodent model of AN, activity-based anorexia (ABA) [57], that elicits rapid body weight loss combined with paradoxical hyperactivity [58] to determine whether psilocybin has differential effects on 5-HTR function in the context of eating disorder pathology. ABA rats exhibit impairments in cognitive flexibility on both reversal learning [59] and attentional-set shifting tasks [60], which is rescued by suppressing cortico-striatal circuitry [61] a key site of psychedelic drug action [62]. Finally, we assessed psilocybin-induced alterations in the abundance of 5-HTR mRNA transcripts in the prefrontal cortex to determine the time-course of effects as well as its impact following the development of the ABA phenotype. Together, these studies reveal specific roles of 5-HT receptor subtypes in enhanced flexible learning after psilocybin and point towards a molecular mechanism that may underpin the efficacy of psilocybin for treating symptoms of AN, including cognitive inflexibility.

## Methods

### Animals and housing

All animals were obtained from the Monash Animal Research Platform (MARP; Clayton, VIC, Australia). To assess direct effects of psilocybin on the development of the ABA phenotype, female Sprague-Dawley rats (*n* = 35 behaviour; *n* = 12 RNAscope) were 6 weeks of age on arrival in the laboratory. Young female rats were used in these studies because they are particularly vulnerable to developing the ABA phenotype, a feature that is incompletely understood but has translational relevance to the increased prevalence of AN in young women. In order to asses cognitive and behavioural phenotypes relevant to AN/ABA, we used separate cohorts of aged matched female Sprague-Dawley rats (total *n* = 168) that commenced training at 7 weeks of age (see Supplementary Table 1 for details). To examine the effects of psilocybin on 5-HTR subtype abundance across a time course, an additional cohort (*n* = 19) of female Sprague-Dawley rats were used, with administration matched to behavioural cohorts at 8 weeks of age. In all cases, animals were group-housed and acclimated to the 12 h light/dark cycle (lights off at 1100 h) for 7 days in a temperature (22-24°C) and humidity (30-50%) controlled room before experiments commenced. Because the behavioural aspects of ABA (i.e. wheel running and food intake) as well as aspects of reinforcement learning are known to fluctuate with the oestrous cycle in female rats [63, 64], a male rat was individually housed in all experimental rooms at least 7 days prior to experimentation in order to facilitate synchronisation of cycling, known as the Whitten Effect [65]. All experimental procedures were conducted in accordance with the Australian Code for the care and use of animals for scientific purposes and approved by the Monash Animal Resource Platform Ethics Committee (ERM 29143).

### Pharmacological compounds

Psilocybin (USONA Institute Investigational Drug Supply Program; Lot# AMS0167) was dissolved in saline and administered at a dose of 1.5 mg/kg. Ketanserin tartrate (Tocris Biosciences, CAS 83846-83-7; 1.5 mg/kg), MDL100907 (volinanserin; Sigma-Aldrich, CAS 139290-65-6; 0.1 mg/kg) and WAY100635 maleate (Tocris Biosciences, CAS 1092679-51-0; 0.5 mg/kg) serotonin receptor subtype antagonists were administered 30 min before psilocybin (or 0.9% NaCl saline control) treatment and all animals only received one combination of psilocybin/saline and one receptor subtype antagonist. Dose selection was based on the literature [46, 66–68]. All drugs were administered intraperitoneally at a 1.0 ml/kg injection volume using a 26-guage needle.

### Activity-based anorexia (ABA)

The ABA paradigm consists of unlimited access to a running wheel and time-restricted food access. At seven weeks of age, rats were individually housed in transparent living chambers with a removable food basket and a running wheel (Lafayette Instruments, IN, USA). Rats were allowed to habituate to the wheel for seven days to determine baseline running wheel activity (RWA). The following day, psilocybin or saline was administered, wheels were locked for 5 h and then reopened. Running activity was recorded by the Scurry Activity Wheel Software (Lafayette Instruments, IN, USA). During the ABA period, food access was restricted to 90 min/day at the onset of the dark phase (1100–1230 h). Running in the hour before the feeding window (1000–1100 h) was considered as food anticipatory activity (FAA). Time-restricted food access persisted for a maximum of 10 days or until rats reached <80% of baseline body weight (ABA susceptibility criterion), at which point they were euthanised with 300 mg/kg sodium pentobarbitone (Lethabarb; Virbac, Australia).

### Home-cage operant learning paradigms

Open-source Feeding Experimentation Devices (Version 3), known as “FED3” [69], were used for home-cage operant testing, fitted with custom built masks. The task wall consisted of two nose-poke ports situated on either side of a pellet magazine where pellets were delivered with a motorised dispenser. Both operant ports and magazines were fitted with infra-red beams to detect nose-pokes and pellet collection, and were controlled by a commercial microcontroller with data displayed on screen for user feedback. An LED strip underneath the nose-poke ports was used as a light cue. The firmware for FED3 devices were written in the Arduino language, modified from the available Arduino library (https://github.com/KravitzLabDevices/FED3) and flashed in sets of operant training menus (https://github.com/Foldi-Lab/LKM_FED3-tasks).

Following light cycle acclimation, rats were individually housed in tinted transparent cages (26 cm W x 21 cm H 47.5 cm D) with *ad libitum* access to water and standard laboratory chow (Barastoc, Australia) throughout. Rats were habituated to sucrose pellet rewards (20 mg, AS5TUT; Test Diet, CA, USA) for two days prior to training. Operant testing was conducted once daily in the home cage for a 3 h session between 12:00–15:00 (early dark phase), which began with two days of magazine training on a “free feeding” schedule in which a pellet was dispensed each time one was removed from the magazine. Subsequently, rats were trained to poke for rewards at fixed ratio (FR) schedules (FR1, FR3, FR5) for 2–5 days each until high accuracy (>80% target responding) was achieved. The target side for all experiments was counterbalanced across each cohort to control for any inherent side bias due to in cage FED3 position. Between animal variability in training performance was always balanced between treatment groups and any animals failing to learn the penultimate training step were removed from the experiment before drug administration (see Supplementary Table 1).

#### Between-session reversal learning task

To test the effects of psilocybin on cognitive flexibility, saline or 5-HTR antagonists (pre-treatment) were administered 30 min prior to either saline or psilocybin (treatment), at the completion of the final FR5 training session. The following day (18 h post-administration) the reward contingencies of the nose-poke ports were reversed (un-cued), and rats underwent 3 days of testing on the reversed FR5 schedule.

#### Fixed and variable ratio schedule training and extinction

To test the effects of psilocybin on suppression of learned FR responding, saline or psilocybin was administered immediately following the final FR5 training session. Over the next 3 days rats underwent extinction testing in which the FED3 was provided as usual except no rewards were delivered regardless of animal activity. To test the effects of psilocybin on training under variable reward schedules, and the long-lasting effects on response suppression, rats were trained to nose-poke at FR1 for 4 days after which saline or psilocybin was administered. The following day rats were trained at variable ratio (VR) schedules of VR5, VR10 and VR20 (two days on each schedule), where the number of target pokes required to deliver a pellet on each trial was randomly selected from 1–5, 6-10 or 11-20, respectively. Subsequently, rats underwent 2 consecutive days of extinction testing, with 24 h access to the FED3 device.

#### Progressive ratio and re-setting task

To test the effects of psilocybin on motivated (effortful) responding, saline or psilocybin was administered at completion of the final FR5 training session and the next day rats underwent a progressive ratio (PR) reinforcement schedule, where the exponential schedule increased according to the formula (5 * e(0.2*n)- 5), where *n* is the trial number, producing response requirements of 1, 2, 4, 6, 9, 12 etc., to the nearest whole number. This was followed by a session at FR5 to reinstate responding and a session during which the PR schedule reset to 1 following any 10 min period of FED3 inactivity, called a re-setting progressive ratio (R-PR) task.

### 5-HT receptor subtype abundance

For detection and quantification of 5-HTR subtypes, rats were administered psilocybin or saline and euthanized with sodium pentobarbitone (Lethabarb 150 mg/kg; Virbac, AU) at a time course (6, 12 or 24 h) post-administration. ABA rats underwent exposure to the model as described above and were administered psilocybin or saline after they had lost at least 15% baseline body weight (15.1-17.6%). Six hours later they were euthanized as above and all rats were transcardially perfused with 200 mL 0.9% saline followed by 200 mL 4% paraformaldehyde in phosphate buffer. Brains were excised and postfixed in 4% paraformaldehyde in phosphate buffer solution overnight at 4 °C, followed by submersion in increasing concentrations of 10%, 20% and 30% sucrose in phosphate buffer solution across 3–4 days. Brains were then sectioned at 15 μm using a cryostat (CM1860; Leica Biosystems) and the medial prefrontal cortex (mPFC) was collected in a 1:4 series. Two mPFC sections per animal, from the same series (spanning from bregma, anteroposterior: +3.2 mm to +2.2 mm), were placed onto SuperFrost Plus slides, and stored at –20 °C until used. The RNAscope^TM^ Multiplex Fluorescent V2 detection reagent kit (Advanced Cell Diagnostics, USA) was used according to manufacturer’s instructions and included specific *in situ hybridisation* probes complementary to the mRNA of the 5-HT1AR (*Rn-Htr1a*; RDS404801) and 5-HT2AR (*Rn-Htr2a*; ADV424551). Detection of mRNA was achieved using Opal™ fluorophore dyes from the 520 (1:500) and 620 (1:750) reagent packs (Akoya Biosciences, USA). Full protocol details are available in Supplementary Methods. Sections were imaged using a widefield microscope (Thunder Imager Live Cell & 3D Assay, Leica Microsystems, Germany) with a PL Fluotar 506007 40x/1.00-0.50-oil Leica objective. The resulting Z-stacks were instantly deconvolved using the integrated Large Volume Computational Clearing deconvolution algorithm (Leica LIGHTNING). The resulting datasets were pre-processed in a custom macro using ImageJ (v1.53t [70]) and analysed with CellProfiler (v4, [71]) using a custom pipeline (see Supplementary Materials) for quantification of nuclear bodies as well as *Htr1a* and *Htr2a* transcripts. Selected sections were analysed further using Imaris software (v9.9, Oxford Instruments) to establish the anatomical location of identified differences in transcript abundance across the cortical layers [72]. The DAPI-channel was used as a mask to define individual cells (cell body selection) and the number of *Hrt1a* or *Hrt2a* puncta surrounding DAPI was analysed using the vesicle detection feature.

### Statistical analyses

Statistical analyses were performed using GraphPad Prism 9.5.1 (GraphPad Software, San Diego, CA, USA). Statistical significance was set at *p* < 0.05, with *p* < .10 considered a trend though not significant. Analyses used were two-tailed unpaired *t* test, one-way and two-way analysis of variance (ANOVA) with Bonferroni’s, Dunnett’s or Sidak’s post hoc multiple comparisons, and a mixed-effects model, chosen appropriately considering the type of data, number of groups, and comparisons of interest. Full details of all statistical tests performed (including group composition) can be found in the Statistics Tables in Supplementary Materials. For RNAscope analyses each individual animal’s data point represents an average value from 4 (individual regions) or 8 (combined regions) sections.

## Results

### Psilocybin improves body weight maintenance in ABA rats

**Fig. 1:**
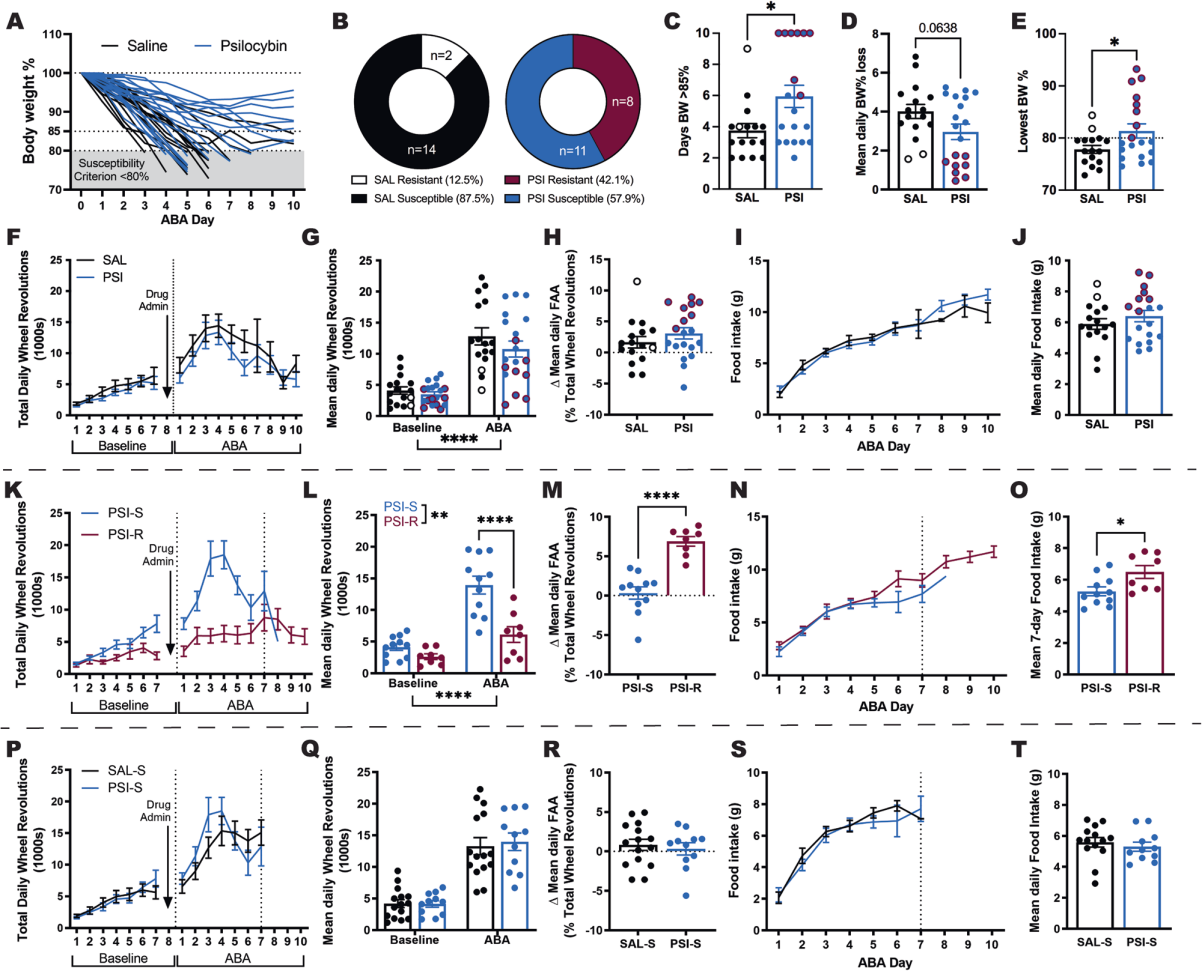
Effects of psilocybin on body weight maintenance in ABA. Weight loss trajectories of individual rats (*n* = 16 saline; *n* = 19 psilocybin) over the 10-day ABA period (**A**) and proportion resistant to weight loss (**B**). Psilocybin facilitated body weight maintenance over 85% for more days (**C**, *t*(33) = 2.508, *p* = 0.0172), with a trend toward lower body weight % loss per day (**D**, *t*(33) = 1.918, *p* = 0.0638) that resulted in attenuation of severe weight loss (**E**, *t*(33) = 2.146, *p* = 0.0394). Total daily wheel revolutions (**F**) increased as expected in ABA (**G**, ABA Phase *F*(1, 33) = 126.5, *p* < 0.0001) but were similar between groups (Treatment *F*(1, 33) = 1.159, *p* = 0.2985) across both baseline (*p* > 0.9999) and ABA (*p* = 0.3089; Interaction *F*(1, 33) = 1.033, *p* = 0.3169) with no difference in the change in proportional running wheel activity in the penultimate hour before food access (**H**, *t*(33) = 0.1.098, *p* = 0.2800). Ninety-minute food intake (**I**) increased similarly across the ABA phase with no difference in mean daily intake (**J**, *t*(33) = 0.9908, *p* = 0.3290). Comparison of only psilocybin treated rats that were susceptible (PSI-S) versus resistant (PSI-R) to ABA highlights the characteristic starvation-induced hyperactivity displayed by PSI-S (**K**) during the first 7 days of exposure to ABA conditions (**L**, ABA Phase *F*(1, 17) = 36.48, *p* < 0.0001; ABA Outcome *F*(1, 17) = 19.01, *p* < 0.0001; Interaction *F*(1, 17) = 17.09, *p* = 0.0047; ABA PSI-S > PSI-R *p* < 0.0001), in contrast to the selective increase of running in anticipation of food access displayed by PSI-R (**M**, *t*(17) = 6.203, *p* < 0.0001), accompanied by diverging food intake trajec*t*ories (**N**) with greater mean 7-day intake by PSI-R (**O**, *t*(17) = 2.577, *p* = 0.0196). Comparison of ABA susceptible rats that received psilocybin (PSI-S) or saline (SAL-S) revealed no differences in the development of starvation-induced hyperactivity (**P**) following the onset of ABA conditions (**Q**; ABA Phase *F*(1, 24) = 126.5, *p* < 0.0001; Treatment *F*(1, 24) = 1.159, *p* = 0.2895; Interaction *F*(1, 24) = 1.033, *p* = 0.3269), selective running in anticipation of food (**R**; *t*(24) = 0.6452, *p* = 05252) or food intake over time (**S**) or on average (**T**; *t*(24) = 0.4783, *p* = 0.6368). Grouped data show mean ± SEM, with individual data points on bar graphs. *p < 0.05, **p < 0.01, ***p < 0.001, ****p < 0.0001. SAL saline, PSI psilocybin, BW body weight, ABA activity-based anorexia, FAA food anticipatory activity, PSI-S psilocybin treated ABA susceptible, PSI-R psilocybin treated ABA resistant. For full statistical analysis details see Fig. 1 Statistics Table.

In order to assess the influence of a single dose of psilocybin on subsequent adaptation to conditions of ABA, psilocybin was administered 24 h prior to the onset of timed food restriction, which facilitated improved body weight maintenance throughout ABA exposure (Fig. 1A) and increased the proportion of animals resistant to the paradigm (Fig. 1B). Psilocybin-treated rats spent significantly more days above 85% of their baseline body weight (*p* = 0.0172; Fig. 1C) and although the reduction in average daily weight loss after psilocybin treatment did not reach statistical significance (*p* = 0.0638; Fig. 1D), psilocybin prevented severe weight loss associated with ABA (*p* = 0.0394; Fig. 1E). This ability to better maintain body weight under ABA conditions was not driven by marked alterations to overall wheel running (Fig. 1F), with psilocybin and saline treated animals running similar amounts during both baseline and ABA phases (baseline *p* > 0.9999, ABA *p* = 0.3089; Fig. 1G) and during the food anticipation period (*p* = 0.2800; Fig. 1H). Similarly, food intake increased over successive days of ABA exposure regardless of treatment (Fig. 1I) and psilocybin did not change the average amount of food consumed across the ABA period (*p* = 0.3290; Fig. 1J). When comparing psilocybin treated rats that were susceptible versus resistant to weight loss, it appeared that psilocybin-induced resistance was not qualitatively distinct from previous work [58, 73], but was similarly defined by both reduced food-restriction evoked hyperactivity (Fig. 1K) that was specific to running during ABA (baseline *p* = 0.7415, ABA *p* < 0.0001; Fig. 1L), increased running in anticipation of food (*p* < 0.0001; Fig. 1M) and increased food intake across days (Fig. 1N) and averaged over the first 7 days of the ABA period (*p* < 0.0196; Fig. 1O). Notably, the only behavioural feature that preceded improved body weight maintenance after psilocybin treatment was wheel running on the day prior to administration (Baseline Day 7; Fig. 1K). We also compared only animals treated with psilocybin or saline that were *susceptible* to developing ABA, to understand whether feeding or exercise outcomes were altered independent of improved weight maintenance. We found that this subgroup of rats treated with psilocybin were indistinguishable from controls on propensity to engage in starvation induced hyperactivity (Fig. 1P) that is elicited by ABA (*p* = 0.2895; Fig. 1Q), running in anticipation of food (*p* = 0.5252; Fig. 1R) or food intake over time (**S**) or on average (*p* = 0.6368; Fig. 1T).

### Psilocybin enhances flexible behaviour in a reversal learning task

**Fig. 2:**
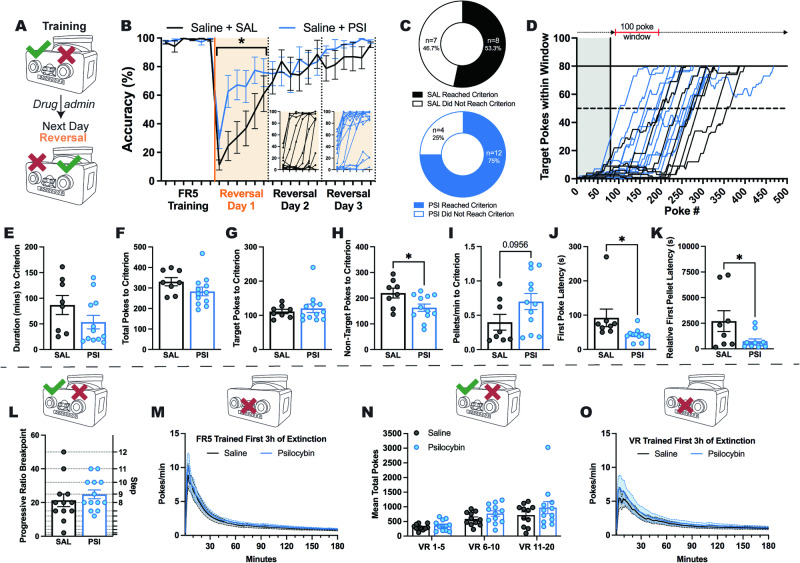
Effects of psilocybin on reversal learning, effortful responding and response suppression. Psilocybin administered after training the day prior to reversal of reward contingencies (**A**) significantly improved accuracy of responding during the initial 3 h reversal session (**B**, Treatment *F*(1, 29) = 5.128, *p* = .0312; 6 x 30 min time bins) and increased the number of rats (**C**) able to reach performance criterion (**D**, 80 target pokes in a 100-poke moving window) on the first day of reversed reward contingencies. While there was no difference in the time (from first poke to poke that achieved criterion; (**E**), *t*(18) = 1.514, *p* = .1474), total pokes (**F**, *t*(18) = 1.536, *p* = 0.1420) or target pokes (**G**, *t*(18) = 0.5614, *p* = 0.5815) required to reach criterion, psilocybin-treated rats required fewer non-target pokes to reach criterion (**H**, *t*(18) = 2.425, *p* = 0.0260), tended to earn rewards faster (**I,** *t*(18) = 1.759, *p* = 0.0956), and were both faster to first engage with the task (time from device access to first poke; (**J**), *t*(18) = 2.307, *p* = 0.0332) and to earn their first reward (time from first poke to earning first pellet; (**K**), *t*(18) = 2.291, *p* = 0.0343). Psilocybin treatment had no effect on breakpoint (pokes required to earn final pellet before 10 min of inactivity) on a classic progressive ratio task (**L,** *t*(23) = 0.7795, *p* = 0.4436), extinction following fixed ratio training (**M**, Treatment *F*(1, 17) = 0.3212, *p* = 0.5783; Interaction *F*(179, 3043) = 0.2623, *p* > 0.9999), goal directed engagement on increasingly uncertain schedules of reinforcement (**N**, Treatment *F*(1, 21) = 1.741, *p* = 0.2013; Interaction *F*(2, 42) = 0.7244, *p* = 0.4906), or extinction following variable ratio training (**O**, Treatment *F*(1, 21) = 0.2681, *p* = 0.6100; Interaction *F*(179, 3759) = 0.3509, *p* > 0.9999). Grouped data show mean ± SEM, with individual data points on bar graphs. *p < .05. SAL saline, PSI psilocybin, FR5 fixed ratio 5, VR variable ratio. For full statistical analysis details see Fig. 2 Statistics Table.

Considering that the ability to maintain body weight during exposure to ABA in rats has been previously linked to improved cognitive flexibility on a reversal learning task [61] and that exposure to ABA conditions impairs reversal learning [59], we hypothesised that the improvements in weight maintenance after psilocybin were associated with improved flexibility in the present study. Psilocybin was administered 18 h prior to reversal of reward contingencies (Fig. 2A), and produced an improvement in response accuracy (*p* = 0.0312; Fig. 2B), evidenced by a rapid shift in responding towards the reversed port and an increase in the proportion of rats that reached performance criterion (Fig. 2C). In order to quantify performance, we used a moving window accuracy (80% accurate, within a 100-poke window) to demonstrate that psilocybin treated rats required fewer trials to learn the task (Fig. 2D). Improved performance after psilocybin was not driven by faster criterion acquisition (*p* = 0.1474; Fig. 2E) or altered total (*p* = 0.1420; Fig. 2F) or target responses (*p* = 0.5815; Fig. 2G), but specifically by reduced responding to the non-target (incorrect) port (*p* = 0.0260; Fig. 2H), indicating psilocybin treatment facilitated learning from negative feedback and faster behavioural adaptation, which was also evident in improved reward efficiency (reduced non-target pokes per pellet; see Supplementary Fig. 1F). While psilocybin did not significantly improve the rate of reward collection (*p* = 0.0956; Fig. 2I), it increased engagement with the reversal task evident in reduced latencies to respond (*p* = 0.0332; Fig. 2J) and win the first reward (*p* = 0.0343; Fig. 2K). To confirm that this improvement was not related to increased effortful responding or response suppression, we tested separate cohorts of rats on progressive ratio (PR), variable ratio (VR) and extinction tasks. Here, we show that psilocybin administration 18 h prior to test did not increase the willingness of rats to expend effort to obtain rewards (*p* = 0.4436; Fig. 2L), the ability to extinguish a previously learned response (*p* = 0.5783; Fig. 2M) or response vigour under uncertain (variable) schedules of reinforcement (*p* = 0.2013; Fig. 2N). Moreover, there was no improvement in response suppression 7 days following psilocybin treatment (*p* = 0.6100; Fig. 2O). See Supplementary Fig. 1 for full session data, including for animals that did not reach performance criterion on the first reversal session.

Because classical PR tasks require the test session to be terminated after a 10 min period of inactivity, and yet psilocybin was shown to increase task engagement in the reversal learning task, we were interested to see if psilocybin also acted to restore responding after periods of inactivity. We tested this in two ways; firstly, by allowing animals access to the operant devices for 3 h using a standard PR schedule and secondly, by implementing a variation of the PR task in which after any 10 min period of inactivity the ratio reset to 1 [re-setting PR (R-PR); see Supplementary Fig. 2A, B]. Breakpoint itself was not different between tasks (all *p*s > .2666; Supplementary Fig. 2C), however, psilocybin increased task engagement specifically during the R-PR session, when increased engagement is considered economical because the effort required to receive a reward is lower. Moreover, psilocybin-induced task engagement was directed rather than arbitrary, with increases in the number of target but not non-target pokes observed when the ratio reset (PR *p* > 0.9999, R-PR *p* = 0.0209; Supplementary 2D, E). None of these changes were observed prior to the first re-setting (i.e. first breakpoint; Supplementary Fig. 2I–M), indicating that experience with the new reward economy was required to elicit increased engagement after psilocybin.

### 5-HT1AR and 5-HT2AR subtype mechanisms differentially drive psilocybin-induced flexible learning

**Fig. 3:**
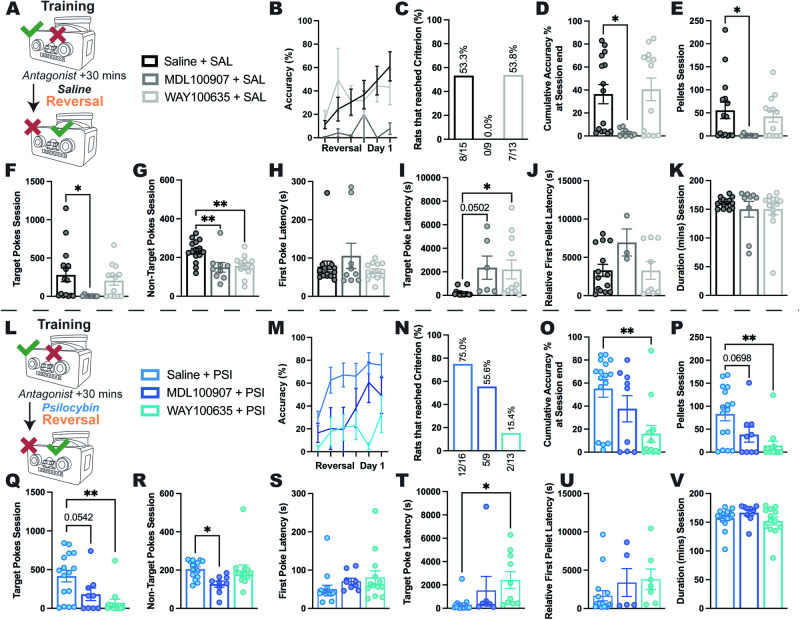
Effects of 5-HT2A and 5-HT1A antagonism on reversal learning in control and psilocybin-treated rats. Following the final training session pre-treatment with either saline (vehicle control), the 5-HT2AR antagonist MDL100907, or the 5-HT1AR antagonist WAY100635, was followed 30 min later by treatment with either saline (**A**) or psilocybin (**L**) before reversal of reward contingencies the following day, with first reversal day performance accuracy highlighted (**B**, **M**, respectively). While 53/5% (8/15) of Saline+SAL rats reached reversal day 1 criterion, 0% (0/9) of MDL + SAL treated rats did so (**C**), showing global impairment compared to Saline+SAL across nearly all outcome measures, achieving significantly lower session accuracy (**D**, SAL + > MDL+ *p* = .0221), earning fewer pellets (**E**, SAL + > MDL+ *p* = .0324), and making fewer target (**F**, SAL + > MDL+ *p* = .0292) and non-target (**G**, SAL + > MDL+ *p* = 0.0048) pokes in the session. While there was no delay in task engagement (time from device access to first poke; **H**, SAL+ vs MDL+ *p* = 0.4572), target poke latency was increased (**I**, SAL + < MDL+ *p* = 0.0502) in the 6/9 rats that made a target poke, and only 3/9 rats earned a single pellet (i.e. made at least 5 target pokes; (**J**) time from first poke to earning first pellet, SAL+ vs MDL+ *p* = 0.1507), even though task engagement duration did not differ (time from first to final poke; (**K**), SAL+ vs MDL+ *p* = 0.6048). Conversely, WAY + SAL resulted in 53.8% (7/13) of rats reaching criterion, nearly identical to Saline+SAL, with these groups being similar across most measures except WAY + SAL having fewer non-target pokes (**G**, SAL + > WAY+ *p* = 0.0041) despite an elongated target poke latency (**I**, SAL + < WAY+ *p* = 0.0244). With 75% (12/16) of Saline+PSI rats reaching reversal day 1 criterion, MDL + PSI treatment produced a moderate decrease to 55.6% (5/9) reaching criterion (**N**), although only a non-significant decrease in accuracy (**O**, SAL+ vs MDL+ *p* = 0.2837), while there was a trend toward fewer pellets (**P**, SAL + > MDL+ *p* = 0.0698) and target pokes (**Q**, SAL + > MDL+ *p* = 0.0542), and a significant reduction in non-target pokes (**R**, SAL + > MDL+ *p* = 0.0210) across the session, with no differences in any latency measures (**S–U**, all SAL+ vs MDL+ *p*s > .2991) or session duration (**V**, SAL+ vs MDL+ *p* = 0.4345). In contrast, WAY + PSI produced severe impairment with only 15.4% (2/13) of rats reaching criterion, with significantly reduced session accuracy (**O**, SAL + > WAY+ *p* = .0024), pellets earned (**P**, SAL + > WAY+ *p* = 0.0015), and target pokes (**Q**, SAL + > WAY+ *p* = 0.0013), and delayed target poke latency (**T**, SAL + < WAY+ *p* = 0.0212, with only 10/13 rats achieving a target poke) compared to Saline+PSI, whilst there were no differences for non-target pokes (**R**, SAL+ vs WAY+ *p* = 0.9497), first *p*oke latency (**S**, SAL+ vs WAY+ *p* = 0.1636), relative first pellet latency (**U**, SAL+ vs WAY+ *p* = 0.2588, although only 7/13 earned a pellet), nor session duration (**V**, SAL+ vs WAY+ *p* = 0.7309). Bar graphs show mean ± SEM with individual data points. *p < 0.05, **p < 0.01. SAL saline, PSI psilocybin, SAL+ saline pre-treatment, MDL + MDL100907 pre-treatment; WAY + WAY100635 pre-treatment. For main ANOVA results and full statistical analysis details see Fig. 3 Statistics Table.

To determine whether psilocybin improved flexibility on the reversal learning task via actions at 5-HT receptor subtypes relevant to AN, selective antagonists to these receptor subtypes were administered 30 min prior to administration of saline (control) or psilocybin and the following day the reward-paired port was reversed (Fig. 3A, L). Analysis of parameters that contribute to accuracy across the first day of reversal learning (Fig. 3B, M) revealed that for control rats, 5-HT2AR antagonism completely abolished reversal learning capability, with 0% of rats administered the 5-HT2AR antagonist (MDL100907) reaching performance criterion, compared to approximately 53% of rats administered the 5-HT1AR antagonist (WAY100635) or saline treatment alone (Fig. 3C). This impairment was driven by all aspects of learning throughout the reversal session, including reduced accuracy (*p* = 0.0221, Fig. 3D), rewards obtained (*p* = 0.0324, Fig. 3E), target pokes (*p* = 0.0292, Fig. 3F) and non-target pokes (*p* = 0.0048, Fig. 3G). Importantly, 5-HT2AR antagonism did not cause an impairment in task initiation, since the latency to respond was equivalent across groups (*p* = 0.4572, Fig. 3H), although it increased the latency to make a target (correct) poke (*p* = .0502, Fig. 3I), suggesting MDL100907 administration prevented control rats from adapting to the new reward rules. Moreover, the impairment elicited by 5-HT2AR antagonism in control rats was not due to reduced willingness to engage in the task, considering there were no significant changes in the latency to receive the first reward (*p* = 0.1507, Fig. 3J) or session duration (*p* = 0.6048, Fig. 3K), however, it should be noted that only three MDL100907 treated control rats ever earned rewards. Conversely, 5-HT1A antagonism did not significantly alter most performance measures throughout the test session (all *p*s > .5609; Fig. 3D–F, H, J, K) but specifically reduced the number of non-target pokes performed (*p* = 0.0041, Fig. 3F) and increased the latency to first target poke (*p* = .0244, Fig. 3I), suggesting administration of WAY100635 allowed rats to learn to the same degree as saline controls with less negative feedback and despite being slower to respond at the initial reversal of reward contingencies.

When combined with psilocybin treatment (Fig. 3L), antagonism of 5-HT1A and 5-HT2A receptors resulted in an opposing pattern of results, with a substantial reduction in 5-HT1AR antagonist (WAY100635) treated animals able to learn the task to criterion (15.4%; Fig. 3N), compared to 55.6% 5-HT2AR antagonist (MDL100907) treated and 75% treated with psilocybin alone. This impairment in reversal learning was demonstrated in reduced accuracy (*p* = 0.0024, Fig. 3O), rewards obtained (*p* = 0.0015, Fig. 3P) and target pokes performed (*p* = 0.0013, Fig. 3Q), however, 5-HT1A antagonism prior to psilocybin treatment did not alter suppression of responding to the previously rewarded (non-target) side (*p* = 0.9497, Fig. 3R) or willingness to initiate a session (*p* = 0.1636, Fig. 3S), although it did increase the latency to poke on the reversed port (*p* = .0212, Fig. 3T). Compared to psilocybin treatment alone, selective 5-HT2A antagonism reduced the number of non-target pokes (*p* = 0.0210, Fig. 3R) but did not significantly alter any other performance measures (all *p*s > 0.0542; Fig. 3O–Q, S-V).

### Psilocybin rescues learning impairments induced by 5-HT2AR antagonism potentially via preferential actions at 5-HT1AR

**Fig. 4:**
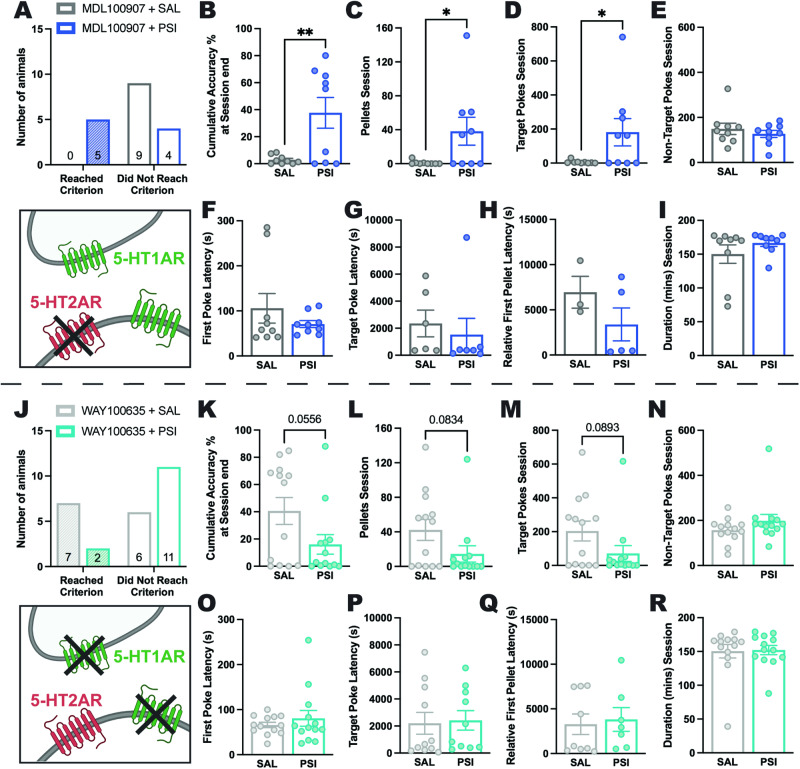
Effects of 5-HT2AR and 5-HT1AR antagonism on psilocybin-induced improvements in reversal learning. Reversal learning following 5-HT2AR antagonism via pre-treatment with MDL100907 (**A**) was completely impaired in saline treated animals (0/9 [0%] reached reversal day 1 criterion) whereas psilocybin treatment prevented this impairment (5/9 [55.6%] reached criterion). Psilocybin treatment following MDL-mediated 5-HT2AR antagonism resulted in significantly greater session accuracy (**B,** *t*(16) = 3.034, *p* = 0.0079), pellets earned (**C,** *t*(16) = 2.255, *p* = 0.385), and target pokes made (**D,** *t*(16) = 2.191, *p* = 0.0436) compared to saline treatment, with no differences for non-target pokes (**E,** *t*(16) = 0.7609, *p* = 0.4578), first poke latency (time from device access to first poke; **F**, *t*(16) = 1.034, *p* = 0.3163), target poke latency (**G**, *t*(11) = 0.5098, *p* = 0.6202), relative first pellet latency (time from first poke to earning first pellet;(**H**), *t*(6) = 1.295, *p* = 0.2428), or session duration (time from first to final poke; (**I**), *t*(16) = 1.133, *p* = 0.2741). The opposite performance pattern was observed following 5-HT1AR antagonism via WAY100635 pre-treatment (**J**), with 7/13 (53.8%) saline treated rats reaching criterion compared with only 2/13 (15.4%) psilocybin treated rats. Although not significant, psilocybin treatment produced a trend toward lower session accuracy (**K**, *t*(24) = 2.011, *p* = 0.0556), fewer pellets earned (**L**, *t*(24) = 1.806, *p* = 0.0834), and target pokes made (**M**, *t*(24) = 1.771, *p* = 0.0893), whilst there was no difference between groups for non-target pokes (**N**, *t*(24) = 1.317, *p* = 0.2001), first poke (**O**, *t*(24) = 0.7925, *p* = 0.4538), target poke (**P**, *t*(19) = 0.1996, *p* = 0.8440), or relative first pellet (**Q**, *t*(14) = 0.3062, *p* = 0.7639) latency, or session duration (**R**, *t*(24) = 0.1337, *p* = 0.8948). Bar graphs show mean ± SEM with individual data points. *p < 0.05, **p < 0.01. SAL saline, PSI psilocybin. For full statistical analysis details see Fig. 4 Statistics Table.

This differential impact of 5-HT2A antagonism is highlighted when comparing performance between saline and psilocybin treated animals that all received MDL100907, a large number of which did not reach performance criterion (Fig. 4A). Whereas selective 5-HT2A antagonism alone (with saline) impaired performance across the board, co-administration of psilocybin rescued impairments in accuracy (*p* = 0.0079, Fig. 4B), rewards earned (*p* = 0.0385, Fig. 4C) and target responses (*p* = 0.0436, Fig. 4D), potentially via preferential actions at the 5-HT1AR. 5-HT2AR antagonism with MDL100907 administration did not cause differential effects on non-target pokes (*p* = .4578, Fig. 4E), or the latencies to first poke (*p* = 0.3163, Fig. 4F), first target poke (*p* = .6202, Fig. 4G) or first reward won (*p* = 0.2428, Fig. 4H) in psilocybin or saline treated rats, nor was the duration engaged in a session (*p* = 0.2741, Fig. 4I) different for psilocybin or saline treated animals that were administered MDL100907. While 5-HT1A antagonism combined with psilocybin substantially reduced the number of rats able to reach performance criterion (Fig. 4J), and induced a trend toward reduced accuracy (*p* = 0.0556, Fig. 4K), instead of a performance impairment per se what seems to be the case is that co-administration of WAY100635 negated psilocybin-induced improvements in reversal learning, with no significant differences in learning measures or response profiles observed between saline and psilocybin treated animals that were administered the 5-HT1AR antagonist (WAY100635) (all *p*s > .0834; Fig. 4L–R). Further supporting a role of 5-HT1A antagonism negating the improvement elicited by psilocybin rather than *impairing* performance is the finding that WAY100635 alone facilitated reversal learning compared to saline alone, by reducing non-target responding (Supplementary Fig. 3F). Intriguingly, co-administration of the mixed antagonist ketanserin impaired performance in saline and psilocybin treated animals to a similar extent, with the notable exception of reducing the session duration for those rats administered saline but not psilocybin (see Supplementary Fig. 4).

### Psilocybin causes a transient shift in the balance of 5-HT1AR and 5-HT2AR mRNA in the prefrontal cortex

**Fig. 5:**
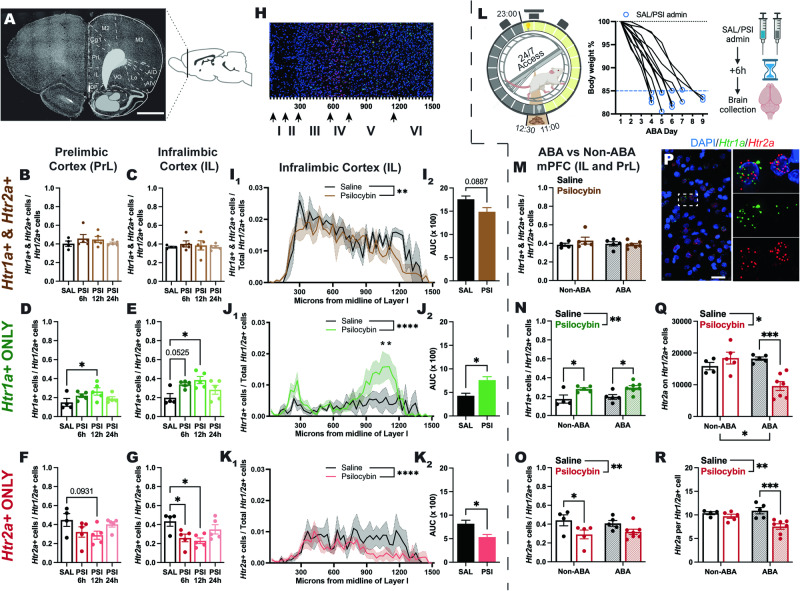
Effects of psilocybin on the expression of *Htr1a* and *Htr2a* transcripts in the mPFC. Coronal section with brain atlas overlay (AP + 3.2 mm from bregma); (**A**) depicting regions of interest (PrL and IL). The proportion of *Htr1/2a*+ cells that were double labelled with *Htr1a* and *Htr2a* was not changed by psilocybin treatment in either the PrL (**B**, *F*(3, 15) = 0.7801, *p* = .5233) or IL (**C**, *F*(3, 15) = 0.2449, *p* = 0.8637). The proportion of *Htr1/2a*+ cells that were exclusively *Htr1a* labelled was increased following psilocybin administration in both the PrL (**D**, *F*(3, 15) = 2.443, *p* = 0.1043, SAL<PSI12h *p* = 0.0500) and the IL (**E**, *F*(3, 15) = 4.277, *p* = 0.0227, SAL<PSI6h *p* = 0.0525, SAL<PSI12h *p* = 0.0103), whilst those exclusively *Htr2a* labelled decreased following psilocybin treatment at a trend level in PrL (**F**, *F*(3, 15) = 2.192, *p* = 0.1314, SAL>PSI12h *p* = 0.0931) and significantly in IL (**G**, *F*(3, 15) = 4.426, *p* = 0.0203, SAL>PSI6h *p* = 0.0335, SAL>PSI12h *p* = 0.0129). The spatial distribution of IL *Htr1/2a*+ cells along the midline from Layer I (**H**) was significantly different for each uniquely labelled cell population (**I**_**1**_ *F*(49, 300) = 10.34, *p* < 0.0001; **J**_**1**_ *F*(49, 300) = 3.549, *p* < 0.0001; **K**_**1**_ *F*(49, 300) = 3.436, *p* < 0.0001). In each case psilocybin treatment also had a significant effect, producing a significantly reduced overall proportion of double labelled cells (**I**_**1**_, *F*(1, 300) = 9.214, *p* = 0.0026) and exclusively *Htr2a* labelled cells (**K**_**1**_, *F*(1, 300) = 19.16, *p* < 0.0001), but a significantly increased overall proportion of exclusively *Htr1a* labelled cells (**J**_**1**_, *F*(1, 300) = 22.38, *p* < 0.0001) accompanied by a significant Distance by Treatment interaction (**J**_**1**_, *F*(49, 300) = 1.459, *p* = 0.0313). AUC was decreased at a trend level for double labelled cells (**I**_**2**_, *t*(6) = 2.030, *p* = 0.0887), significantly increased for exclusively *Htr1a* labelled cells (**J**_**2**_, *t*(6) = 3.102, *p* = 0.0211) and significantly reduced for exclusively *Htr2a* labelled cells (**K**_**2**_, *t*(6) = 3.097, *p* = 0.0212). A separate cohort of animals underwent ABA induction, were administered either saline or psilocybin when they reached <85% baseline body weight, and culled ~6h later (when bodyweight had dropped to close to ~80% in most cases; **L**). The proportion of mPFC *Htr1/2a*+ cells that expressed both *Htr1a* and *Htr2a* was not effected by psilocybin administration nor ABA exposure (**M**, Treatment *F*(1, 17) = 0.5663, *p* = 0.4620; ABA Exposure *F*(1, 17) = 0.4685, *p* = 0.5029; Interaction *F*(1, 17) = 1.208, *p* = 0.2871), whereas psilocybin significantly increased or significantly decreased the proportion of exclusively *Htr1a* labelled (**N**, Treatment *F*(1, 17) = 15.50, *p* = 0.0011; Non-ABA SAL < PSI *p* = 0.0298, ABA SAL < PSI *p* = 0.0206) or *Htr2a* labelled (**O**, Treatment *F*(1, 17) = 9.038, *p* = 0.0079; Non-ABA SAL > PSI *p* = 0.0463) cells, respectively, in a generally consistent and ABA independent manner (all ABA exposure and interaction *p*s > 0.4607). *Htr1a* (green) and *Htr2a* (red) expression on distinct cell populations in the mPFC (**P**) identified through DAPI (blue). The absolute number of *Htr2a* transcri*p*ts associated with mPFC *Htr1/2a*+ cells (**Q**) was significantly altered by psilocybin (*F*(1, 17) = 4.587, *p* = 0.0470), ABA exposure (*F*(1, 17) = 5.098, *p* = 0.0374), and their interaction (*F*(1, 17) = 15.26, *p* = 0.0011), such that psilocybin treatment significantly reduced *Htr2a* copy number specifically in the ABA brain (ABA SAL > PSI *p* = 0.0005). This pattern was mostly replicated by the number of *Htr2a* copies per mPFC *Htr1/2a*+ cell (**R**, Treatment *F*(1, 17) = 12.12, *p* = 0.0029; ABA Exposure *F*(1, 17) = 2.048, *p* = 0.1705; Interaction *F*(1, 17) = 5.606, *p* = 0.0300), with a significant reduction in the density of *Htr2a* specifically following psilocybin treatment after ABA induction (ABA SAL > PSI *p* = 0.0007). Grouped data show mean ± SEM, with individual data points on bar graphs (except AUC). Values are the average of 4 (PrL and IL) or 8 (mPFC) sections per animal. *p < 0.05, **p < 0.01, ***p < 0.001, ****p < 0.0001. AP anterior-posterior, SAL saline, PSI psilocybin, PrL prelimbic cortex, IL infralimbic cortex, AUC area under the curve, mPFC medial prefrontal cortex (PrL and IL combined); *Htr1/2a*+ cells expressing *Htr1a* and/or *Htr2a*; ABA activity-based anorexia. Scale bars for (**A**) 2 mm and (**P**) 30 µm. For full statistical analysis details see Fig. 5 Statistics Table.

To examine whether a change in the abundance of 5-HTR subtypes in medial prefrontal cortex (mPFC) was elicited by psilocybin, which could explain the differential effects of psilocybin on reversal learning after pharmacological blockade of the 5-HT1A vs 5-HT2A receptor subtypes, we performed RNAscope on cortical sections (Fig. 5A) collected 6, 12 and 24 h after psilocybin treatment.

Psilocybin had no effects on the proportion of cells positive for both *Htr1a* and *Htr2a* transcripts in either the prelimbic (*p* = 0.5233, Fig. 5B) or infralimbic (*p* = 0.8637, Fig. 5C) subregions of the mPFC, but significantly increased the proportion of cells exclusively positive for *Htr1a* in both subregions (prelimbic; *p* = 0.0500, Fig. 5D, infralimbic; *p* = 0.0103, Fig. 5E) at 12 h post-administration. This was matched with complementary reductions elicited by psilocybin in the proportion of cells exclusively positive for *Htr2a* in both subregions, although this did not reach statistical significance in the prelimbic cortex (*p* = 0.0931, Fig. 5F) and was evident at both 6 h and 12 h timepoints in infralimbic cortex (6 h; *p* = 0.0335, 12 h; *p* = 0.0129, Fig. 5G). We further examined the anatomical localisation of *Htr1a* and *Htr2a* positive cells across cortical layers in the infralimbic cortex (Fig. 5H) at 12 h post-administration, to show an overall reduction in double positive cells after psilocybin treatment (*p* = 0.0026, Fig. 5I_1_) that did not reach significance when analysed as an area under the curve (AUC; *p* = 0.0887, Fig. 5I_2_). What was clear, however, was that the respective increase and decrease in the proportion of cells exclusively positive for *Htr1a* or *Htr2a* 12 h after psilocybin treatment was specifically localised to cortical Layer V (*Htr1a p* < 0.0001, Fig. 5J_1_, *p* = 0.0211, Fig. 5J_2_; *Htr2a p* < 0.0001, Fig. 5K_1_*p* = 0.0212, Fig. 5K_2_), which corresponded to 900-1200 µm distance from the midline of Layer I (see Fig. 5H).

To determine whether psilocybin had similar effects on *Htr1a* and *Htr2a* expression in the context of weight loss and feeding pathology relevant to AN, we compared transcripts from the saline and 6 h psilocybin treated rats (non-ABA) to rats that had exhibited substantial weight loss after exposure to ABA conditions (and brains collected 6 h after psilocybin administration) (Fig. 5L). Main effects of psilocybin identified in non-ABA rats were recapitulated for ABA rats, with no changes in the number of double labelled cells (*p* = 0.4620, Fig. 5M) but complementary increases in *Htr1a* (*p* = 0.0011, Fig. 5N) and decreases in *Htr2a* (*p* = 0.0079, Fig. 5O) positive cells, indicating similar consequences of psilocybin treatment occurred in the ABA brain. Multiple comparisons revealed that the increase in the number of *Htr1a* positive cells elicited by psilocybin was stronger in ABA rats than non-ABA rats (non-ABA; *p* = .0298, ABA; *p* = 0.0206, Fig. 5N), while the decrease in *Htr2a* positive cells was weaker (non-ABA; *p* = 0.0463, ABA; *p* = 0.2102, Fig. 5O). However, additional changes in the abundance of *Htr2a* transcripts (Fig. 5P) were observed following weight loss under ABA conditions, whereby psilocybin elicited a substantial reduction in the overall number of *Htr2a* transcripts (*p* = 0.0005, Fig. 5Q) and in the number of *Htr2a* transcripts per cell (*p* = 0.0007, Fig. 5R) in the mPFC of ABA rats, that was not evident in non-ABA rats. Further analyses of changes in *Htr1a* and *Htr2a* expression over the 24 h time course and effects of exposure to ABA are provided in Supplementary Fig. 5.

## Discussion

Clinical trials evaluating the safety and efficacy of psilocybin in people with AN have been ongoing since 2019, with the first pilot study recently reporting that it improves eating disorder symptoms in some individuals, but not others [74]. Psilocybin may have transdiagnostic efficacy [18, 31, 40] through several mechanisms relevant to the pathology of AN, including actions on the serotonergic system [39] and cognitive flexibility [22]. However, the details of how such mechanisms are altered by psilocybin in the context of AN remains unknown. Here, we show that psilocybin improves body weight maintenance in the ABA rat model and enhances cognitive flexibility in a reversal learning task by both reducing perseverative responding and promoting task engagement when reward contingencies are initially reversed. That psilocybin did not elicit changes in motivated responding (PR) or response suppression (extinction) following the same training and drug administration protocol suggests a selective improvement in adaptive cognition in the face of changing rules.

Further, we demonstrate that psilocybin-induced improvements in reversal learning performance were not dependent on binding to the 5-HT2AR, because co-administration of the selective 5-HT2AR antagonist (MDL100907) did not significantly alter performance measures. Instead, the action of psilocybin at the 5-HT1AR was required for improved cognitive flexibility, whereby improvements in reversal accuracy and engagement were abolished when psilocybin was co-administered with the selective 5-HT1AR antagonist (WAY100635). This finding is complicated by the relatively fast-acting effects of psilocybin observed on *Hrt2a and Hrt1a* transcription in the mPFC, which indicates that psilocybin rapidly and transiently alters the balance of the cellular machinery required to support receptor binding in this region associated with cognitive flexibility [49]. In such a way, the differential effects of 5-HT2A and 5-HT1A antagonism on reversal learning after psilocybin may reflect functional interactions between these two receptor subtypes that depend on serotonin availability during the post-acute (~24 h) administration period [75]. These outcomes also call into question reports of the necessity of 5-HT2A binding for “therapeutic” outcomes of psilocybin in animal models, particularly those that use the non-selective antagonist, ketanserin. Not only does ketanserin bind multiple serotoninergic and non-serotonergic receptors but it also only blocks ~30% of 5-HT2AR in the rat cortex [76]. It is plausible, therefore, that partial blockade with ketanserin shifts the binding of psilocybin to other 5-HTR subtypes, including 5-HT1A, which may explain the acute improvement in reversal learning after ketanserin alone previously reported in rats [38].

The finding that psilocybin administration specifically prevented severe weight loss in ABA rats is critical in light of the evidence that lower body mass increases the risk for fatal outcomes in AN [77, 78]. That psilocybin treatment did not have overall effects on feeding or exercise independently is unsurprising considering that psilocybin does not alter feeding or energy balance in mouse models of obesity [79] and supports the proposal that the therapeutic effects of psilocybin for anorexia nervosa are more likely driven by adaptive cognition than through metabolic alterations [53]. In line with this, resistance to weight loss after psilocybin was associated with all aspects of behavioural adaptation to ABA conditions (i.e. increased food intake, reduced compulsive running and increased motivated running), which we have previously shown to be linked with improved cognitive flexibility in ABA rats [59, 61]. While we only observed trend level reductions in overall body weight loss after psilocybin administration, the treatment group is clearly comprised of two distinct subgroups – those that respond to psilocybin with improved weight outcomes and those that are indistinguishable from controls. This divergence in response profiles exists in multiple clinical populations, where between 40-80% of individuals report therapeutic benefits of psilocybin assisted psychotherapy at follow-up, dependent on trial parameters [16, 74, 80]. Response variation was also seen in the pilot study of psilocybin in people with AN, with clinically significant improvements seen in 4/10 participants [74]. The effects of psilocybin on ABA and cognitive flexibility were not assayed in the same subjects in the present study, due to confounds associated with using food rewards in a model that is typified by disturbed feeding behaviour and dysregulated reward processing [81]. However, considering the specific effects of psilocybin on perseverative behaviour during reward reversal observed, perhaps those individuals (humans or rats) who respond to psilocybin with positive body weight outcomes represent a subgroup whose profile is typified by rigid patterns of thought and behaviour. This information could guide the clinical application of psilocybin to those individuals demonstrating high rigidity. In a similar vein, the observation that rats that responded to psilocybin with improved weight outcomes demonstrated lower levels of running during the baseline phase (i.e. prior to treatment and the onset of ABA conditions; see Supplementary Fig. 6) points to the intriguing potential that psilocybin may be more efficacious in animals (and possibly people) that already have a lower propensity to engage in excessive exercise.

The translational relevance of administering psilocybin prior to ABA exposure in this study, instead of after the establishment of anorectic phenotypes (as is the case for the clinical situation) requires some elaboration. Because of the ethical requirement to remove animals from the ABA paradigm when they reach the weight loss criterion that deems them “susceptible”, it is not possible to intervene at this point to attempt to improve outcomes. We did, however, delay administration after a specific duration of ABA exposure (2 days) or after a specific amount of weight loss (15%) in separate cohorts of rats. This intervention produced worse outcomes for *both* saline and psilocybin treated rats, likely due to the additional stress associated with handling and injection at this critical point of the ABA paradigm (see Supplementary Methods & Supplementary Fig. 7). It is also important to recognise the short generation time for the ABA phenotype compared to the often long and protracted pathogenesis of AN [82] that may underscore differences in the timing and nature of impairments in cognitive flexibility between human and rodent. Whereas cognitive inflexibility may exist prior to onset of AN symptoms and contribute to the development of the condition, it does not predict susceptibility to ABA but develops coincident with weight loss in rats [59]. Thus, the effects of prior administration of psilocybin on weight maintenance has relevance for the specific type of inflexibility that develops in the context of eating pathology in the rat model. In both cases (human and rat) further research is required to understand how psilocybin might elicit meaningful changes in body weight maintenance over the long term, what neurobiological mechanisms differentiate “responders” from “non-responders” and whether the same mechanisms underpin the effects of psilocybin on body weight maintenance and cognitive flexibility [83].

The specific focus in the present study on the involvement of 5-HT2A and 5-HT1A receptor subtypes was based in the evidence from imaging studies that AN is associated with decreased 5-HT2A and increased 5-HT1A binding in cortical regions [23, 24]. The finding that psilocybin has the same main effects on the number of cortical cells exclusively positive for the *Htr1a* and *Htr2a* transcripts in animals that had been exposed to ABA conditions is important for the clinical application of psilocybin for AN, and suggests that at least some of the neurobiological effects of psilocybin are unchanged by the development of AN-relevant symptoms. Notably, psilocybin treatment in ABA rats was associated with an augmented increase in the number of cells exclusively positive for *Htr1a* transcripts and an additional reduction in the abundance *Htr2a* transcripts (i.e. number of transcripts per cell) that was not seen after psilocybin treatment in rats that were naïve to ABA. This suggests that in the context of AN-associated symptoms, the actions of psilocybin on cellular activity in the mPFC is more inhibitory in nature, which could indeed be therapeutically relevant in light of the evidence that AN is associated with exaggerated cortical activity [84]. Perhaps then, it is this additional boost of inhibitory tone elicited by psilocybin in ABA rats that allows them to better adapt to the experimental conditions when psilocybin treatment is administered prior to onset.

The overall influence of psilocybin on the number of mPFC cells that exclusively express *Htr1a* and *Htr2a* transcripts is also relevant for understanding the involvement of activity in this brain region for cognitive inflexibility in ABA rats. If one considers the large majority (60–75%) of mPFC Layer V cells (where the effects of psilocybin were localised) that express these mRNAs are pyramidal (glutamatergic) cells, the net effect of psilocybin during this 12 h window would be hyperpolarisation of the mPFC, via both increasing the inhibitory 5-HT1AR and decreasing the excitatory 5-HT2AR machinery. This aligns with our previous work, in which chemogenetic suppression of mPFC projection neurons could both prevent weight loss in ABA and improve flexibility on a reversal learning task [61]. However, *Htr1a* and *Htr2a* transcripts are also present on at least two classes of GABAergic interneurons in this cortical region, complicating the interpretation of effects of psilocybin on excitatory output [85]. Moreover, 5-HT1AR are expressed both pre- and post-synaptically, with differential effects of binding on serotonergic transmission [86, 87]. Finally, the alterations observed at a transcriptional level does not preclude other mechanisms such as protein degradation or changes in receptor cycling [88, 89] from being involved in the serotonergic consequences of psilocybin treatment.

With respect to the specific improvement in reversal learning elicited by psilocybin as a mechanism to explain improved body weight maintenance during ABA, it is notable that the reduced perseverative responding when reward contingencies were reversed was also driven by a subpopulation of “responders”. This raises the possibility that individual differences in baseline serotonin signalling may underlie responses to psilocybin treatment, as proposed by the inverted “U-shaped” dose-effect relationships reported for many active compounds and their relation to cognitive function [90]. If adaptive cognition requires an appropriate balance between 5-HT1A and 5-HT2A receptor function [49], our molecular findings suggest that individuals exhibiting elevated 5-HT1AR function (or for that matter reduced 5-HT2AR function) may not respond positively (since further elevation or reduction elicited by psilocybin would push them into the tail ends of the inverted “U”). It is also important to note, in light of the recent observation that psilocybin, administered acutely, did not facilitate flexibility [38], that there are important methodological differences that may explain this discordance. Specifically, we examined effects of psilocybin post-acutely, using a single administration paradigm, and the reversal learning task used in the present study relied on action-outcome learning rather than Pavlovian cue-outcome learning. Performance on this task is also dependent on the incentive salience of rewards to elicit appropriate responding, with psilocybin-induced improvements observed in reversal task engagement, leading to faster receipt of the first (unexpected) reward. This demonstrates the potential of psilocybin to alter the explore/exploit trade-off common in reinforcement learning, where the subject has the option of maximizing reward based on its current information (exploitation) or by accumulating more evidence (exploration) [91] and may improve the balance between the two for more effective adaptation.

One of the most intriguing issues related to the actions of psilocybin in the brain is the means via which it changes neuronal morphology and function to exert its effects. The canonical pathway through which psilocybin is proposed to promote plasticity (and presumably therefore flexible learning) is through binding to the 5-HT2A receptor, an act that elicits a “glutamate surge” through rapid increases in neuronal excitability [29, 30]. The dendritic and synaptic changes that occur downstream may or may not be related to this surge of glutamate since psilocybin induced structural plasticity was still observed in the presence of ketanserin [46]. It is convenient to focus the actions of psilocybin at 5-HT2AR located in the PFC because of their requirement for the subjective (psychedelic) effects [43], however, this view discounts the abundant expression of 5-HT2A in other brain regions relevant to learning and memory, including the hippocampus, claustrum and striatum [92]. The results of the present study suggest that improvements in flexible learning after psilocybin are not mediated by binding to the 5-HT2A receptor, but that selective 5-HT2A antagonism impaired learning in all animals, an effect that was partially restored with co-administration with psilocybin. A possible explanation for these results is that while 5-HT2A receptor function is required for reversal learning, it only partly supports the cognitive enhancing effects of psilocybin. A major challenge is in understanding the role of the 5-HT1A receptor in mediating learning outcomes, especially since firing activity of 5-HT neurons in the dorsal raphe nucleus is controlled by pre-synaptic expression of 5-HT1AR, where binding inhibits serotonin release [92]. We show that 5-HT1A antagonism did not affect the ability to reach performance criteria or obtain reward in controls, but preferentially impaired learning improvements elicited by psilocybin. Taken together, this highlights the 5-HT1AR as an important target mediating the effects of psilocybin on cognitive flexibility [92].

The key outcomes of this study underline the fact that animal studies are required for understanding the mechanisms that underlie the therapeutic efficacy of psilocybin because they allow detailed interrogation of behaviour and brain function in the absence of effects of expectancy. It is important to note that female animals were used exclusively in these studies and that psilocybin has been shown to have effects that differ based on biological sex in rats and mice in terms of behaviour [93], regional brain reactivity [94] and structural neuroplasticity [46]. Future studies should aim to examine how psilocybin influences serotoninergic tone via other (i.e. non-5-HT2A) known 5-HT binding targets in both male and female animals. In addition, real-time longitudinal monitoring of 5-HT activity (i.e. with fibre photometry) could be employed in combination with pharmacological tools to shed light on the 5-HT2AR drivers of weight loss in ABA rats, by measuring dynamic changes as animals progress through the paradigm. This would allow precise differentiation between the effects of psilocybin on susceptible versus resistant subgroups, information that would be particularly relevant for the clinical application of psilocybin in individuals with AN. Additionally, examination of the interaction between serotonergic and dopaminergic mechanisms that influence the way that inflexible patterns of thought and behaviour relate to food reward, aversion, and avoidance [95] should be a focus for future research. That psilocybin has direct actions on the dopamine system in humans [96] and rats [51] has long been known, but surprisingly paid little attention [97], even though the interaction between serotonin receptor binding and dopamine release is well established [98]. The proposal to study dopaminergic effects of psilocybin is brought into sharper focus by recent evidence of brain-wide activation of the dopamine system by ketamine [99] and that dopamine D2 receptor blockade attenuates the psychedelic-induced head-twitch response [100]. These considerations, in concert with the new data presented here, will provide a better understanding of a mechanistic framework of psilocybin actions in the brain, insight that will provide greater confidence in the potential therapeutic use of psilocybin for conditions such as AN. This is an important and arguably necessary step towards including psilocybin in the armoury of tools to treat mental health disorders.

## Supplementary information

Supplementary Materials

## Data Availability

All data supporting the findings of this study are available within the paper and its Supplementary Information.
